# Comparing public support for nuclear and wind energy in Washington State

**DOI:** 10.1371/journal.pone.0284208

**Published:** 2023-04-26

**Authors:** Azusa Uji, Jaehyun Song, Nives Dolšak, Aseem Prakash

**Affiliations:** 1 Graduate School of Law, Kyoto University, Kyoto, Japan; 2 Faculty of Informatics, Kansai University, Osaka, Japan; 3 School of Marine and Environmental Affairs, University of Washington, Seattle, Washington, United States of America; 4 Department of Political Science, University of Washington, Seattle, Washington, United States of America; Shenzhen University, CHINA

## Abstract

The proliferation of country and state-level net zero-emission commitments, rising energy costs, and the quest for energy security in the wake of the Ukraine crisis have renewed the debate about the future of energy sources. As opposed to elite discourse, the energy policy preferences of the public remain less explored. While many public opinion surveys report preferences for a specific type of clean energy, there is less work on understanding choices among different types. We explore whether support for nuclear over wind energy at the state level depends on how people assess the impact of these energy sources on health, local jobs, landscape disruption, and the stability of the electricity supply. Importantly, we seek to understand where people physically reside (and their experience of existing energy possibilities) might influence their energy policy preferences. We estimate multiple regression models with OLS with our original survey data of a representative sample of Washington residents (n = 844). We find that the physical proximity to existing energy facilities *does not* influence support for nuclear over wind energy. However, this support is shaped by the importance respondents attach to health (-), jobs (-), landscapes (+), and supply stability (+) dimensions of energy source. Moreover, the physical proximity to existing energy facilities moderates the importance respondents attach to these dimensions.

## Introduction

Energy is the key driver of economic growth. Policy discussions on energy transition focus on balancing the imperatives of economic growth with climate protection, national security, and equity [1]. However, how the public evaluates trade-offs between different energy policy goals remains relatively underexplored. An understanding of public perceptions is important because public opinion is a key input in shaping the political feasibility of energy policy choices.

In this paper, we focus on how the public evaluates tradeoffs between different zero-emission energy sources and what factors might influence these trades offs. In particular, we are interested in public perceptions about the role of nuclear energy in the decarbonization of the electricity sector, in relation to wind energy. Many environmental groups continue to remain skeptical of, if not opposed to, nuclear energy and advocate the transition to wind and solar instead [2]. While both nuclear and wind are zero-emission energy sources, they pose different economic and ecological consequences. Suppose a state is planning to create additional electricity capacity or replace existing capacity with new ones. Might people living in physical proximity to wind farms or nuclear plants, who might have experienced their pros and cons for health, local jobs, or landscape aesthetics, support wind (nuclear) energy expansion at the state level? We explore these questions in the context of the state of Washington, which has committed to carbon-neutral electricity by 2030 and 100 percent clean electricity by 2045 [3]. Washington state is closing down the Centralia coal power plant, which accounts for the state’s 10% greenhouse emissions [4]. At the same time, the state is also removing hydroelectric dams because they adversely affect the salmon population. Moreover, with the increasing incidence of drought, the hydroelectricity generation (in Washington as well as in the Colorado river basin) has begun to show variation, necessitating the need to plan for backups or alternative sources of energy.

Washington state’s energy demand is also rising due to increases in population and the state’s decision to ban the sale of new gasoline-powered cars by 2030. The bottom line is that Washington state needs to construct new zero-emission electricity facilities or expand existing facilities. Though the state is actively considering wind and nuclear, it is not clear what factors drive public preferences for nuclear over wind or vice versa. For reference, in 2021, both wind and nuclear contributed to about 8% of Washington’s electricity needs, while solar contributed only 0.04%. Other energy sources are hydro (66%), natural gas (13%), and coal (4.5%) [5].

Might individual support for new nuclear and wind capacity at the *state level* be influenced by their physical proximity to existing wind and nuclear facilities? To assess this subject, we identify four local economic and ecological impacts of existing facilities: human health, local jobs, landscape aesthetics, and stability in electricity supply. Nuclear power produces stable 24x7 electricity and creates permanent, well-paying local jobs, and has a small footprint on local aesthetics. However, it exposes the local population to radiation risks. Contrast this with wind facilities that do not pose serious health hazards (although some mistakenly fear radiation), generate lease payments for local communities, and create jobs during construction. However, wind turbines clash with landscape aesthetics. Moreover, wind turbines have an intermittency issue: generate electricity only when there is wind flow.

Washington State has one nuclear plant, the Columbia Generating Station (in addition to the Department of Defense’s Hartford facility) located in Richland, the Tri-City area of Eastern Washington. The state has several utility-scale wind facilities, with a cumulative installed capacity of 3,396 MW, located predominantly in Eastern Washington as of 2021 [5]. Broadly, Eastern Washington, across the Cascades, is rural and conservative (with some urban pockets). At the same time, Western Washington, which lies between the Cascade Mountains and Puget Sound, is urbanized and liberal [6]. While much of the electricity is consumed in Western Washington, it tends to be generated in Eastern Washington, be it hydro, nuclear, or wind. In a recent debate in the state legislature, Rep. Mark Klicker (R-Walla Walla) noted that “Eastern Washington communities are burdened with generating renewable energy, while more populated areas west of the Cascades use most of that energy” [7]. Indeed, many local counties have enacted ordinances to restrict the construction of renewable energy facilities. For example, a newly proposed wind project for the Horse Heaven Hills in Benton County faced local opposition mainly because of the visibility of wind turbines [8]. Eventually, Benton County commissioners unanimously opposed the project in March 2021 [9].

More broadly, the debate on meeting the net-zero emission goals while managing local opposition to new renewable energy facilities is playing out across U.S. states [10–12] and countries [13–15]. Instead of assessing public support for a given energy source, we examine how the public chooses between two zero-emission sources, nuclear and wind, by extending the NIMBY/YIMBY discussions in a new direction. We are interested in exploring how varying levels of physical proximity to existing energy facilities might shape public preferences for new nuclear and wind capacity *at the state level* (as opposed to respondents’ neighborhoods, which the not-in-my-backyard or NIMBY and yes-in-my-backyard or YIMBY research has examined).

Our survey of a representative sample of Washington residents (n = 844) finds that physical proximity to existing energy facilities *does not* influence support for nuclear over wind energy at the state level. Moreover, the support is not influenced by whether respondents reside in Eastern or Western Washington, which represents different types of political cultures. Broadly confirming existing research, the support for nuclear over wind is shaped by the importance respondents attach to health (-), jobs (-), landscapes (+), and supply stability (+) dimensions of energy supply. Moreover, the physical proximity moderates the importance respondents attach to these dimensions in their support of nuclear over wind.

While the literature has investigated public support for specific energy projects, scholars have paid less attention to the public’s valuation of trade-offs among different energy sources, an important issue because there are multiple pathways to decarbonization involving different energy mixes. We highlight that the public does not assess the impacts of energy choices on society uniformly. This suggests that to assess public support for a specific energy mix, policymakers should carefully tailor their communication of the benefits and costs of different energy policy options to different audiences. We made a theoretical contribution by showing that NIMBY/YIMBY insights, originally developed to understand local opposition to unwanted local facilities, are valuable for understanding public support for energy policy choices at the state level, outside the backyard.

## Literature review

Climate change is an outcome of the modern industrial economy’s dependence on fossil fuels. Decarbonization of the energy sector is therefore critical to address the climate crisis. The policy challenge is that most benefits of decarbonization have features of a global public good and raise concerns about free-riding by countries. Since the 1990s, various international treaties and agreements have sought to foster global collective action, curb free riding in decarbonization and motivate countries to pursue zero greenhouse gas emission targets. The 2015 Paris Agreement is a landmark in global climate action because, for the first time, developing countries such as China and India (which were exempt from mandatory emission reductions under the 1997 Kyot0 Protocol but in recent years, have emerged as leading carbon emitters) announced targets for emission reductions. There are many pathways to the decarbonization of the energy sector, involving different zero-emission sources with different ecological and economic impacts. For example, whereas some view nuclear energy as critical for decarbonization, others fear its adverse health impacts as well as the challenges associated with the disposal of radioactive waste. Scholars have employed advanced econometric models and techniques to o investigate the environmental footprint, human development, environmental costs, and ecological footprint of nuclear energy across countries, including the US [16–20]. Other studies have calculated the impact of the energy transition on the ecological footprint in manufacturing countries [21] and the impact of green energy investment on environmental quality (e.g., exploitation of natural resources), the economic and ecological impacts of energy [22]. While these scholars have successfully explored the “objective” impact of energy. However, how the public evaluates these impacts, which shape their preferences for energy choices, remains relatively understudied. Exceptions include Bergmann et al. (2006) who focus on how Scottish respondents value different dimensions of renewable projects, such as wildlife, air pollution, employment, or electricity prices [23]. In their study of Finnish respondents, Kosenius and Ollikainen (2013) examine the benefits and costs of renewable technologies in terms of biodiversity loss, local jobs, carbon emissions, and household electricity bills, and their impact on people’s willingness to pay for renewable energy (wind, crop, wood, and hydro) [24].

In the US context, while there is impressive literature examining support for a specific type of zero-emission energy, there is less work on how the public chooses between different types of zero-emission sources. Noblet et al. (2015) found that, in Maine, citizens support land-based wind more than deep-water offshore or tidal energy [25]. With the nationwide survey, Roe et al. (2001) found that consumers expressed different levels of willingness to pay for residential electricity service by different fuel mixes [26]. Likewise, based on the survey conducted in Delaware, Borchers et al. (2007) found that public willingness to pay for participation in green energy electricity programs depends on renewable energy types [27]. Whereas these studies advanced our understanding of public preferences for renewable energy, how the public evaluates different economic and ecological impacts of different energy sources remains unanswered. To bridge the gap, we seek to understand public assessment of the trading-off between different energy sources and how it might be conditioned by respondents’ physical proximity to an existing energy source.

## Theory -NIMBY/YIMBY

NIMBY (not-in-my-backyard) scholars note that the siting of locally “unwanted” projects, the ones that pose risk to the local community (such as landfills, mining, quarrying, hazardous waste facilities, or prisons) or generally impose costs, provoke local opposition [28–32]. In the context of energy projects, the literature reports this opposition in the context of wind [33, 34], solar [35], and nuclear [36–41]. This opposition extends to energy infrastructure as well. For example, Nelson et al. (2018) find that concerns about health risks and property values motivated opposition to the siting of a new high-voltage transmission line in California [42].

However, scholars have also noted instances of “YIMBY,” in which local communities support “undesirable” industries in their backyards to secure local economic benefits or due to partisan identities [43–45]. For example, Thorpe (2015) finds support for prisons in the rural areas of the American South as these prisons are an important contributor to economic activity [46]. Uji et al. (2021) find that support for restarting nuclear plants when local jobs and low emissions of nuclear energy are highlighted [47]. Jerolmack and Walker (2018) find that rural residents in Pennsylvania support fracking in their backyards [48]. In sum, while NIMBY/YIMBY is motivated by concerns about local costs/benefits from new local projects, the debate reveals how communities value costs and benefits in different ways.

We extend the idea of NIMBY/YIMBY in two ways. First, might experience with existing local projects also inform individual preferences about *state-level* (non-local) energy policy? After all, individuals might have experienced specific harms/benefits first-hand, and this experience might influence their support for state-level energy policy choices. Second, we apply NIMBY/YIMBY, originally developed with a focus on a single project or a single energy source, to assess how the public evaluates one energy source *in relation* to another. This is an important issue because while individuals might oppose nuclear energy per se, they might moderate their opposition if they recognize that they will need to live with coal instead. Thus, individuals often make implicit trade-offs among multiple energy sources, which might be influenced by their familiarity with local energy facilities. In this situation, while individuals might recognize the risks or costs posed by an energy source, they might still support it because they perceive the risks posed by another energy source to be higher. Likewise, although individuals might appreciate the benefits of a given energy source, they might still support some other energy source because they perceive its benefits to be higher.

## Hypotheses on energy policy preferences in Washington State

We focus on the state of Washington, which is often regarded as a climate leader. The state is actively debating how to meet rising energy demand and, at the same time, reduce greenhouse gas emissions and retire hydroelectricity. Democrats control both the chambers of the state legislature and the office of the governor. In addition, both Senators are Democrats, and in the Presidential elections, the state has voted for the Democratic nominee since 1988. Yet, the state voted down two statewide carbon tax referendums: Initiative 732 in 2016 which secured 41% vote, and Initiative 1631 in 2018 which secured 43% vote [49–51]. The 2016 referendum was for a revenue-neutral carbon tax. Although the 2018 vote was for a revenue-positive carbon tax, none of the monies were to be devoted to supporting wind, solar, or nuclear energy. Thus, while zero-emission energy has not been on the ballot, Washington state residents, who are considered to be strongly pro-environment, have defeated pro-climate ballot initiatives in the past. Environmentalists, therefore, pursued pro-climate policy through the state legislature. In 2020, the state enacted a Cap and Invest law (along with other climate bills such as the Clean Fuel Standard), avoiding the political challenges of enacting carbon taxes through ballot initiatives [52].

We recognize that the politics of new taxes tends to differ from that of state-level policy ideas such as creating different types of zero-emission capacities. Yet, historically, environmental groups have had an uneasy relationship with nuclear energy [53]. Some groups such as Greenpeace (2007) remain opposed to nuclear energy. In the German cabinet (which has both Greens and Liberals), the Green party wants nuclear reactors to be decommissioned while the Liberals want their operational life to be extended [54]. Given the strong pro-environment sentiment in Washington state (notwithstanding the defeat of two referendums), we expect Washington residents to support wind energy over nuclear energy.

H1: Respondents are more supportive of wind over nuclear energy at the state level.

Washington state also hosts a nuclear power plant, which is located alongside the Hanford Department of Defense. Moreover, several rural counties have passed a slew of local ordinances to disallow wind energy facilities. Hence, we expect support for wind and nuclear energy to vary across the state. One might argue that the crucial driver of energy policy preferences is their first-hand negative exposure to energy facilities in their physical proximity [12], even when new facilities may not be located in the neighborhood. If so, individuals who have been negatively affected by energy facilities in their neighborhood might support alternative energy.

H2a: Respondents residing closer to the Richland nuclear facility support wind over nuclear at the state level.

H2b: Respondents residing closer to wind facilities support nuclear over wind at the state level.

Support for nuclear over wind might depend on how respondents weigh various benefits and costs of these energy sources. We consider four factors in this regard: health impacts, local jobs, landscape disruption, and stable electricity supply. Nuclear energy is often viewed in negative terms due to the possibility of radiation leaks and the disposal of nuclear waste. Indeed, Hanford’s Department of Defense facility is notorious for poor waste disposal facilities. The federal government has spent billions of dollars in remedial action for cleaning up this facility [55, 56]. While the Columbia Generating Station Richland facility is distinct from the Department of Defense Hanford facility, both are located within the same nuclear complex. Thus, the Hanford effect regarding radioactive leaks might affect how people evaluate the health consequences of new generation capacity at the Richland facility.

The second dimension of interest is the facility’s impact on the local economy. While both nuclear and wind facilities generate jobs, nuclear facilities tend to provide long-term jobs while wind provides temporary jobs, mainly during the construction phase [57, 58]. Therefore, we expect that those who value local jobs will express more support for nuclear over wind.

The third dimension pertains to the disruption in the local landscape [12]. Some might find wind turbines to be ugly and not fitting well with the rural landscape, perhaps even imparting an industrial feel to them. Nuclear plants, in contrast, tend to have a smaller footprint and are less problematic in terms of their aesthetics. And in any case, any expansion of nuclear in Washington state will probably take place within the Richland facility. Thus, people might favor nuclear over wind for landscape aesthetic reasons.

The fourth dimension pertains to the stability of the electricity supply. Wind turbines generate electricity only when there is a sufficient wind flow, the so-called intermittency problem [59]. Indeed, intermittency requires that wind systems have backups (typically, natural gas in the United States) or are integrated with other energy sources which compensate for their intermittent generation. Energy storage is another option for addressing the intermittency of wind and solar [60]. Nuclear, in contrast, generates electricity 24X7. Thus, individuals who value a stable electricity supply (for personal or professional reasons) will support nuclear over wind. Based on the above discussion, we propose the following hypotheses.

H3a: Respondents attaching high importance to health effects support wind over nuclear at the state level.

H3b: Respondents attaching high importance to local jobs support nuclear over wind.

H3c: Respondents attaching high importance to preserving local landscape support nuclear over wind at the state level.

H3d: Respondents attaching high importance to stable electricity supply support nuclear over wind at the state level.

The impact of the above four mechanisms–health, economy, landscape, and supply stability–on respondents’ support for nuclear over wind might be conditioned by their proximity to existing nuclear or wind facilities. This is because respondents, who live closer to these facilities, are more familiar with or sensitive to the pros and cons of choosing specific energy sources. Then, they can better associate each mechanism with either type of energy, which implicitly influences their energy policy preferences. For example, among respondents attaching high importance to the health effects, those living in the vicinity of nuclear plants might favor wind over nuclear, being more sensitive to the risks of high radioactive leaks. On the other hand, those living in the vicinity of wind facilities might recognize that wind energy is relatively harmless firsthand and therefore support it over nuclear. Alternatively, wind energy harms the local landscape and compromises the ruralness of their areas, or the moving of wind turbines causes shadow flickers, which annoy local households.

H4a: Respondents attaching high importance to health issues will prefer wind over nuclear at the state level when they live closer to the Richland power plant.

H4b: Respondents attaching high importance to health issues will prefer wind over nuclear at the state level when they live closer to wind facilities.

Among respondents attaching high importance to local jobs, those living near nuclear plants might support nuclear over wind. This is because these local communities appreciate the fact that the construction of nuclear plants provides ample local jobs based on their experience, while they are less sure about the job creation effect of wind facilities. For the same reason, those living near wind facilities expect more jobs from wind facilities firsthand but are unsure about the case of nuclear power plants. This might lead them to support wind over nuclear.

H5a: Respondents attaching high importance to jobs will prefer nuclear over wind at the state level when they live closer to the Richland nuclear power plant.

H5b: Respondents attaching high importance to jobs will prefer wind over nuclear at the state level when they live closer to a wind facility.

Regarding the third mechanism, landscape aesthetics, respondents attaching high importance to preserving the local landscape and living in the vicinity of wind facilities might support nuclear over the wind. This is because they learned about the disruption of the local landscape by wind facilities through their own experience. On the other hand, those living near nuclear facilities do not associate landscape aesthetics with wind facilities so strongly. Thus, for them, landscape aesthetics are not a strong reason for opposing wind.

H6a: Respondents attaching high importance to landscape aesthetics will not show a preference for wind over nuclear at the state level when they live closer to the Richland nuclear plant.

H6b: Respondents attaching high importance to landscape aesthetics will prefer nuclear over wind at the state level when they live closer to wind facilities.

Regarding the fourth mechanism, stable electricity supply, the public will equally associate stable electricity with nuclear energy regardless of where they live. This is because they can enjoy stable electricity generated by nuclear power plants irrespective of their geographical location. Therefore, proximity to either nuclear or wind facilities will probably not condition support for nuclear over wind.

H7: Respondents attaching high importance to stable electricity supply will prefer nuclear over wind at the state level irrespective of where they live.

## Data and methods

### Data

We administered a survey to a sample of adult citizens who are 18 or older in Washington State (N = 1200). After the approval from the Human Subjects Division of our institution (#STUDY00014049), we pre-registered our survey (https://osf.io/hfyka?view_only=f3ebf29d821d4d0bb9005788fd286528). We employed the services of a commercial firm, Lucid, to administer the survey between November 18–22, 2021. At the start of our survey, we obtained written informed consent from survey participants. As shown in Table 1, we break down our sample by region, gender, age, education, and party identification. Our sample is representative of Washington state’s population in terms of age, gender, regional salience, education, and party identification while females under 75 are slightly over-representative. However, the potential selection bias behind this does not affect our analysis because, in our regression models, we include gender and age as covariates across models [61].

**Table 1:** Representativeness of our sample.

Gender and Age (American Community Survey 2019)				
Region	Gender	Age	In Sample (%)	ACS (%)
Entire	Female	18–24	8.1	5.5
	Female	25–54	35.0	25.8
	Female	55–74	17.2	14.6
	Female	75 -	1.4	4.4
	Male	18–24	3.0	6.0
	Male	25–54	23.2	26.8
	Male	55–74	9.7	13.7
	Male	75 -	2.5	3.2
Eastern	Female	18–24	8.1	6.7
	Female	25–54	33.3	24.0
	Female	55–74	23.9	14.8
	Female	75 -	1.3	4.7
	Male	18–24	3.0	7.0
	Male	25–54	20.9	24.9
	Male	55–74	5.8	14.2
	Male	75 -	2.6	3.6
Western	Female	18–24	8.0	5.2
	Female	25–54	35.6	26.3
	Female	55–74	14.6	14.6
	Female	75 -	1.5	4.3
	Male	18–24	3.0	5.7
	Male	25–54	24.1	27.3
	Male	55–74	10.8	13.5
	Male	75 -	2.5	3.1
Education (American Community Survey 2019)				
Region	Education		In Sample (%)	ACS (%)
Entire	High school incomplete or below		2.8	7.7
	High school		18.5	21.6
	College (no degree)		33.3	31.8
	4-years college		30.2	23.8
	Graduate school		15.2	15.1
Party Identification (American National Election Studies 2020)				
Region	Party		In Sample (%)	ANES (%)
Entire	Democrat		45.0	42.9
	Independent		32.0	36.4
	Republican		23.0	20.7

We are most interested in understanding support for nuclear energy in relation to wind energy, how their support might be influenced by the pros and cons of these energy sources, and by geography (specifically, distance from the closest existing wind facility or the Richland nuclear facility). Our survey is structured as follows (the full text is available in Text A4 in the S1 Appendix). Participants first read about the energy challenge in the State of Washington arising from the increasing electricity demand, the decarbonization goal, and the move to shut down coal and hydroelectric facilities. Next, we presented a table showing the pros and cons of nuclear and wind power (See Table 2).

**Table 2:** Pros and cons of nuclear and wind.

	Pros	Cons
Nuclear	• Generates electricity whenever needed (24 hours and 7 days a week) • A large number of permanent local jobs	• Communities living near nuclear plants face a radiation risk • Nuclear waste needs to be stored safely
Wind	• Farmers and rural landowners can earn income by leasing their land to wind farms • New jobs, especially during the construction.	• Wind turbines clash with the rural landscape, which could hurt land values • Electricity generated only when there is wind

After the attention check questions, we then asked respondents to indicate their support in terms of what percentage of new electricity capacity should be wind or nuclear. We provided two slider scales (0–100%) for wind and nuclear so that respondents could choose the combination of nuclear and wind to meet Washington’s future electricity needs. For example, if the respondent supported 34% wind, then the respondent also automatically supported 66% nuclear. This way, respondents could think about the salience of wind and nuclear *together*. Our dependent variable is the *difference* in support level between nuclear and wind: that is, the level of nuclear support (%) minus the level of wind support (%). This substantively represents the desired percentage of the new electricity capacity of nuclear in relation to wind. The variable ranges between -100 and 100: 0 means that respondents support nuclear and wind equally, and positive and negative signs respectively mean “support nuclear more than wind” and “support wind more than nuclear.” In the above example, our dependent variable takes the value of 32% (66–34). With this measure, we cannot distinguish whether people support both or neither when the difference is 0% (50–50). However, this does not hamper our aim to capture the *relative support* between nuclear and wind, as opposed to the support for one by itself.

To explore the mechanisms motivating respondents’ choices about the nuclear-wind energy mix, we asked about the importance they attach to specific issues (also listed in the information table). These are the health effects of electricity generation and waste disposal (*Health*), local jobs and economic benefits (*Job*), disruption of the rural landscape (*Landscape*), and reliability of electricity supply (*Supply*). The objective is to understand *why* respondents support a specific mix of nuclear energy in relation to wind energy.

We also included questions about other factors that could influence respondents’ support for nuclear or wind energy. Specifically, drawing on the question about environmental attitudes posed in World Values Survey, we asked respondents which statement is closer to their point of view: “protecting the environment should be given priority, even if it causes slower economic growth and some loss of jobs,” or “economic growth and creating jobs should be the top priority, even if the environment suffers to some extent”. We also asked about respondents’ trust in the federal Nuclear Regulatory Commission, Washington State Department of Health, and nuclear plant operators. Given the public concerns about safety, nuclear energy is subject to a host of regulations at multiple levels, including by the NRC. These sorts of safety concerns are less relevant for wind energy. Hence, we did not include any NRC equivalent for wind energy. Finally, we asked standard demographic questions on gender, age, income, education, race, religion, and political ideology.

Importantly, we asked for information about respondents’ residential zip codes to capture the distance from existing energy facilities.

### Model construction

To test our hypotheses, we constructed two multiple linear regression models. Model 1 assesses the association between proximity to existing energy facilities (*Dist_wind and Dist_nuclear*) and the support for nuclear over wind (Hypotheses 2ab). Variables *Health*, *Job*, *Landscape*, *and Supply* capture the importance respondents attach (on a scale of 1–4, where 1 reflects “not important at all”, and 4 reflects “very important”) to nuclear/wind’s health effects, economic impact, landscape disruption and reliability in electricity supply (Hypotheses 3abcd).

We controlled for theoretically important variables that could influence the outcome variable. In addition, we controlled for variables, including demographic factors that could influence both energy preferences and the proximity to energy facilities, given a potential selection bias caused by the reverse effect of energy preference on where respondents reside [62]. Such variables are environmental attitude *(Env)*, trust in bodies regulating safety in the nuclear industry (*Trust_reg*, *Trust_health*, and *Trust_plant*), and whether respondents reside in an urban or rural area (*Urban_rural*). Additionally, we also explored a model by controlling for whether respondents reside in Eastern or Western Washington.

Our model controls for demographic variables: *Gender*, *Age*, *Income*, *Education*, *Race*, *Religion*, *and Party Identification*. Finally, the zip code information also helps us to identify whether the respondents reside in an urban or rural whose climate and energy politics often tend to diverge [63–65]. Thus, *Urban_rural*, included as a control in our models, is a binary variable reflecting whether respondents live in urban or rural areas. Specifically, following the U.S. Census, we coded the county in which a zip code is located as urban when its population is greater than 10,000, and rural otherwise.

In Model 2, we introduce interaction terms to test Hypotheses 4ab, 5ab, 6ab, and 7. We explore how the support for the nuclear-wind energy mix (the dependent variable) varies with respondents’ valuation of *Health*, *Job*, *Landscape*, *and Supply* dimensions, conditional upon respondents’ proximity to an energy facility. Thus, our model includes eight theoretically important interaction terms between each issue (*Health*, *Job*, *Landscape*, *and Supply)* and the distance variable (*Dist_wind and Dist_nuclear*). *Dist_nuclear and Dist_wind* respectively represent the distance (in miles) of the respondent’s county to the Richland nuclear power plant or the closest wind facility.

#### Model 1

$${Nucle\hat{ar-}Wind}_{i}=\alpha +\beta_{1}{Dist\_nuclear}_{i}+\beta_{2}{Dist\_wind}_{i}+\beta_{3}{Health}_{i}+\beta_{4}{Job}_{i}+\beta_{5}{Landscape}_{i}+\beta_{6}{Supply}_{i}+\gamma X_{i},$$

where ***γ*** and ***X***_***i***_ are coefficients vector and demographic variable vector of respondent *i*, respectively.

#### Model 2

$${Nucle\hat{ar-}Wind}_{i}=\alpha +\beta_{1}{Dist\_nuclear}_{i}+\beta_{2}{Dist\_wind}_{i}+\beta_{3}{Health}_{i}+\beta_{4}{Job}_{i}+\beta_{5}{Landscape}_{i}+\beta_{6}{Supply}_{i}+\beta_{7}{Dist\_nuclear}_{i}\cdot {Health}_{i}+\beta_{8}{Dist\_nuclear}_{i}\cdot {Job}_{i}+\beta_{9}{Dist\_nuclear}_{i}\cdot {Landscape}_{i}+\beta_{10}{Dist\_nuclear}_{i}\cdot {Supply}_{i}+\beta_{11}{Dist\_wind}_{i}\cdot {Health}_{i}+\beta_{12}{Dist\_wind}_{i}\cdot {Job}_{i}+\beta_{13}{Dist\_wind}_{i}\cdot {Landscape}_{i}+\beta_{14}{Dist\_wind}_{i}\cdot {Supply}_{i}+\gamma X_{i},$$

where ***γ*** and ***X***_***i***_ are coefficients vector and demographic variable vector of respondent *i*, respectively.

### Estimation techniques

**Fig 1:**
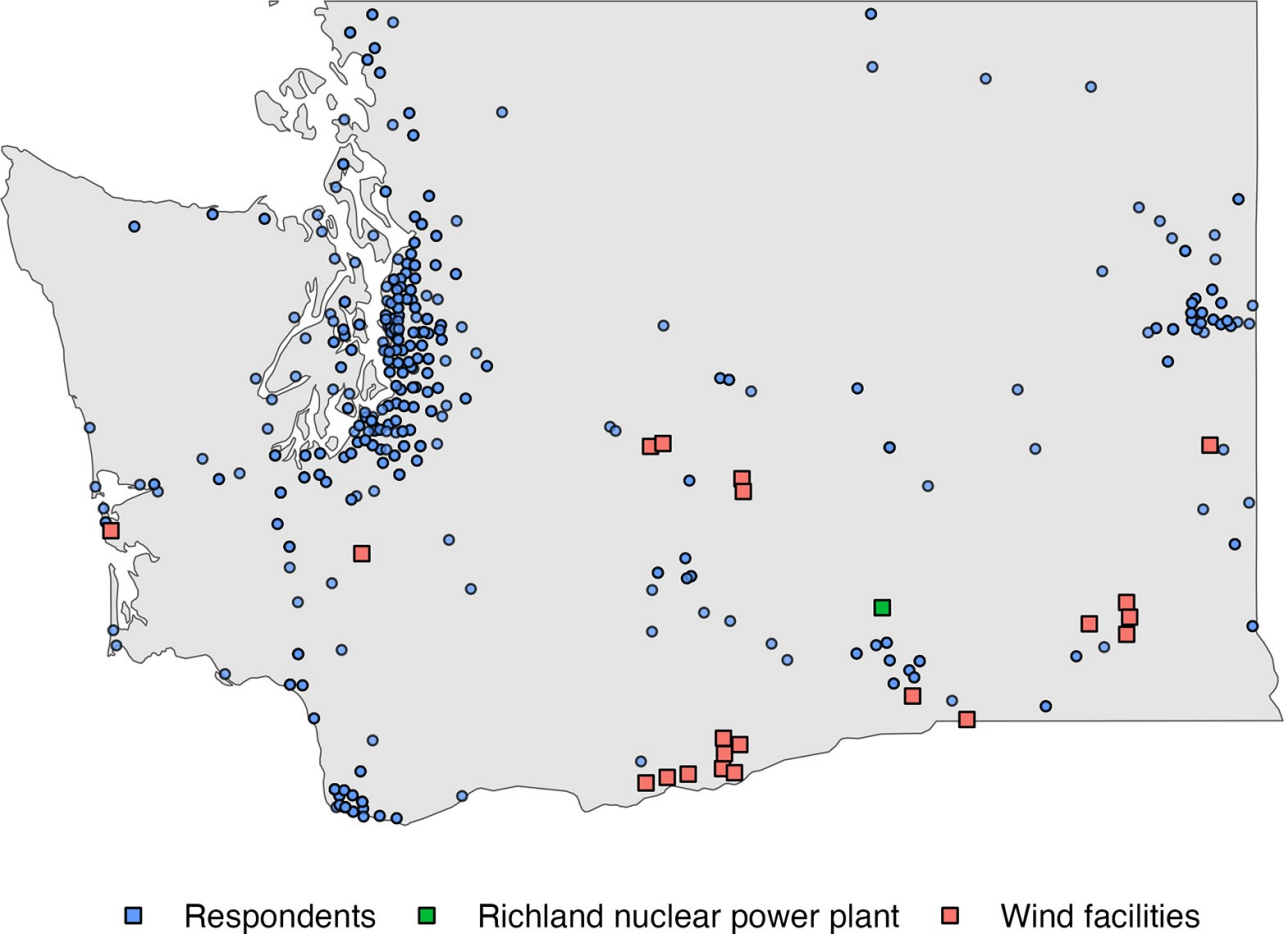
The location of the nuclear power plant, wind facilities, and respondents.

*Dist_nuclear and Dist_wind* are calculated as follows. Based on the collected respondents’ ZIP code information, we obtained the coordinates where respondents live, using DataIntegration.Info (https://dataintegration.info). Based on U.S. Energy Information Administration, we have geocoded the existing nuclear and wind facilities in Washington state. Then, using the *geosphere* package in R, we assessed respondents’ distance (WGS84 ellipsoid) in miles to the nuclear power plant and the closest wind facilities and whether this proximity might play a role in their choice of a particular energy mix. Fig 1 maps the location of the Richland nuclear power plant and 21 existing wind facilities in Washington state and where respondents are located.

We excluded some cases with missing values and non-attentive respondents from the original sample for analysis. We administered the survey to 1200 respondents, of which 330 (27.5%) selected the option, “Don’t want to answer” for a series of questions. In addition, we dropped inattentive respondents, who could not correctly answer any of the three attention check questions included after the introduction text. Consequently, the usable sample for the statistical analysis is 844. We recognize the debate on the potential bias by excluding inattentive respondents. As reported in Table A1 in the S1 Appendix, our substantive findings do not change even when we include both attentive and inattentive respondents. Since our dependent variable, which ranges between -100 and 100, is continuous, we employ the OLS estimator to estimate Models 1 and 2. With Model 1, we estimate the coefficients of explanatory and control variables to see their direct effect on public preferences for energy mix. Then, with Model 2, we estimate the average marginal effect of *Health*, *Job*, *Landscape*, *and Supply*, that is, how these effects on support for energy mix are conditioned by how far respondents live from existing energy facilities. Given our relatively small sample size, we use 90% confidence intervals to test our hypotheses.

## Results

**Fig 2:**
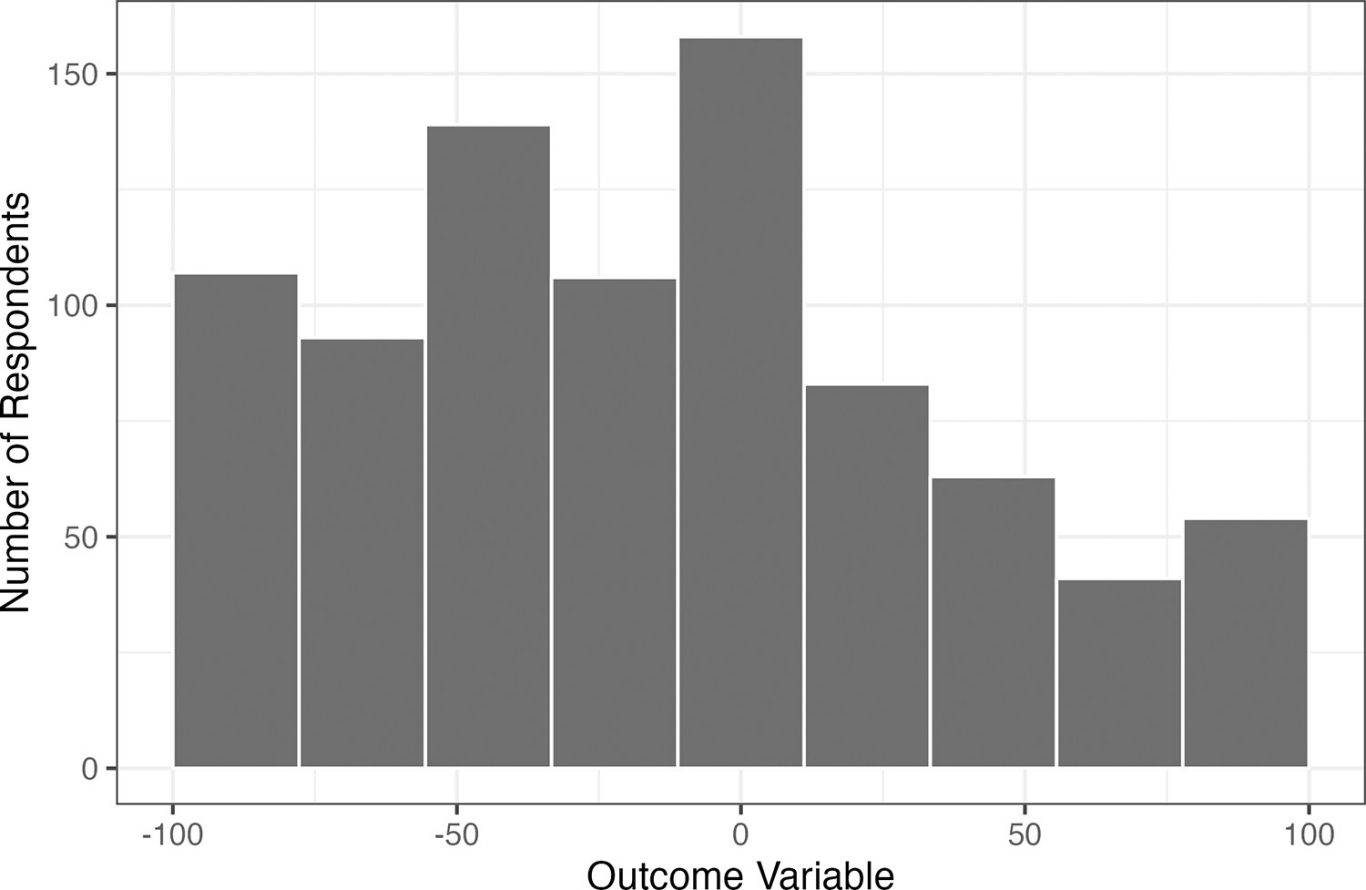
The overall support for nuclear in relation to wind.

First, we illustrate the distribution of the dependent variable: nuclear support (%)–wind support (%). Fig 2 shows the number of respondents who support different combinations of nuclear and wind. The mean value is -14.538, and the 95% confidence interval is [-18.013, -11.063] (p<0.001). This means that residents of Washington State support wind more than nuclear energy, which supports Hypothesis 1.

### Independent effects of geography and pros and cons

We first present the key result of Model 1 in Table 3. Full results, including race and religion, are available in Table A2 in the S1 Appendix.

**Table 3:** Result of Model 1.

	Model 1		
	Coef.	SE	
Dist_nuclear	-0.029	0.044	
Dist_wind	0.063	0.066	
Health	-5.896	1.649	***
Job	-5.159	1.916	**
Landscape	4.205	1.515	**
Supply	9.262	2.514	***
Trust_reg	4.370	2.309	^+^
Trust_health	-10.090	2.016	***
Trust_plant	17.889	2.096	***
Env	-17.761	3.940	***
Urban_rural	4.818	4.783	
Male	8.901	3.262	**
Age (Year of birth)			
1946 to 1964	-13.570	8.253	
1965 to 1996	-18.840	8.210	*
After 1996	-15.426	9.318	^+^
Income			
$40,000 - $69,999	-0.485	4.029	
$70,000 - $104,999	-3.851	4.400	
$105,000 - $159,999	-9.189	5.253	^+^
$160,000 -	1.055	6.577	
Education			
High school (incomplete)	91.985	44.529	*
High school	98.998	43.602	*
College (no degree)	95.667	43.535	*
4 yrs. college	96.635	43.558	*
Grad. school	102.391	43.585	*
Party Identification			
Democrat	-7.371	4.302	^+^
Independent	1.092	4.164	
Intercept	-93.862	46.343	*
*N*	844		
Adj. *R*^2^	0.323		

Note

^+^: p < 0.1

*: p < 0.05

**: p < 0.01

***: p<0.001

Does physical proximity to existing energy facilities shape respondents’ preferences for nuclear over wind? We find that neither the distance to the existing Richland nuclear facility nor the closest wind facility are statistically significant. Therefore, Hypotheses 2a and 2b are not supported.

Does respondents’ evaluation of the pros and cons of energy sources (*Health*, *Job*, *Landscape*, *and Supply)* influence their support for nuclear over wind? Those attaching high importance to stable electricity supply and landscape preservation are more supportive of nuclear over wind (H3cd are supported), while respondents attaching importance to health are less supportive of nuclear over wind (Hypothesis 3a is supported). Contrary to Hypothesis 3b, we find concerns about local jobs drive support for wind over nuclear, rather than vice-versa, as we had hypothesized. We speculate that the media attention on the economic payoff for renewable energy might have created a perception of the high levels of local economic benefits of wind facilities.

Regarding the control variables, *Env* has a statistically significant negative effect on nuclear support, which suggests that those with pro-environment preferences support wind over nuclear. This is consistent with the finding of the literature [25, 66]. *Urban_rural* is not statistically significant, which means that urban residents do not differ from rural residents regarding their support for nuclear over wind. This contrasts with the previous findings that people living in rural areas are less concerned about climate change [67] or findings of distinct preferences over the social and environmental impacts of renewables [23]. Positive and statistically significant effects of *Trust_reg* and *Trust_plant* indicate that those who trust the Federal Nuclear Regulatory Commission and nuclear plant operators support nuclear over wind, probably because they feel that there is adequate oversight about nuclear safety issues. On the other hand, those who trust the Washington State Department of Health support wind over nuclear. Arguably, respondents probably do not think that the Department of Health monitors radiation risks and hence their risk concerns about nuclear are not alleviated even when they trust this state-level Health agency.

Regarding individual-level attributes, the younger generation (below 56 years old) is more supportive of wind over nuclear. Likewise, Democrats are more supportive of wind over nuclear than Republicans. This is consistent with the literature that finds that Democrats/liberals are more willing to pay for renewable energy and a carbon tax than Republicans/conservatives [66, 68, 69]. On the other hand, male and high-educated people support nuclear over wind. This is consistent with previous findings that female, higher-educated, or young people are more supportive of renewable energy [23, 24]. We did not find a systematic association between income level and support for nuclear over wind, which is consistent with Bergmann et al. (2006) and Allen Wolters et al. (2020) [23, 66]. In addition, religion and race do not affect the level of support for nuclear over wind.

### Interaction effects of geography and pros and cons

**Fig 3:**
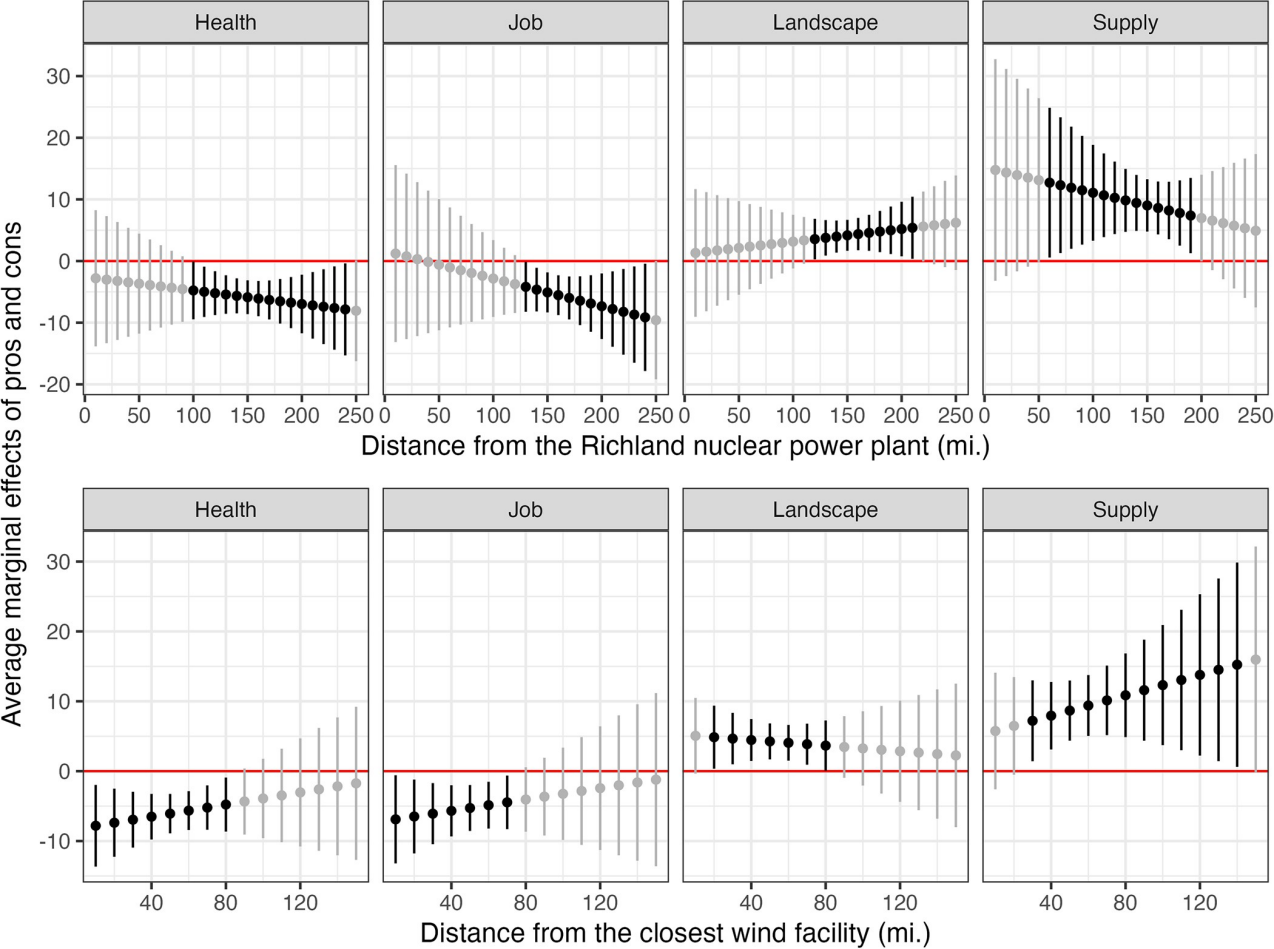
Average marginal effects of pros and cons. Note: Points indicate the average marginal effects and lines represent 90% confidence intervals. Statistically significant average marginal effects are displayed in bold.

Does the proximity to the existing energy facilities moderate how respondents evaluate the pros and cons of nuclear and wind, and eventually their support for nuclear over wind at the state level? To explore this issue, in Model 2, we interact *Health*, *Job*, *Landscape*, *and Supply* variables with respondents’ distance from the Richland nuclear power plant and existing wind facilities. The upper and lower panels of Fig 3 respectively show the average marginal effect of *Health*, *Job*, *Landscape*, *and Supply* on support for nuclear over wind with 90% confidence intervals by different levels of distance to the nuclear power plant and the closest wind facility. When we adopt 95% confidence intervals, the range of distance where average marginal effects are statistically significant becomes slightly smaller, but our main substantive results hold. The regression table for the result of Model 2 is available in Table A3 in the S1 Appendix.

The upper panel of *health* shows that health concern reduces support for nuclear over wind only when the respondents live at a distance *greater* than 100 miles from the Richland nuclear facility. This is contrary to our expectations that health concerns will diminish support for nuclear over wind when respondents live in proximity to the nuclear power plant (Hypothesis 4a is not supported). This may be because living with a nuclear power plant eases rather than intensifies their concerns about the health risks of nuclear power. Unlike Chornobyl or Fukushima nuclear power plants, the Richland power plant has never caused a nuclear accident. On the other hand, the lower panel of *health* is characterized by the statistically significant negative average marginal effect when the distance to the closer wind facility is less than 80 miles. This is the evidence that people living closer to wind turbines support wind over nuclear for the health concern, which supports Hypothesis 4b.

The upper and lower panels of *Job* indicate that respondents attaching high salience to local jobs and living closer to wind facilities are less supportive of nuclear over wind (Hypothesis 5b is supported). However, we find no such effect for respondents living close to the Richland nuclear power plant (Hypothesis 5a is not supported). This may be because people living close to the Richland nuclear power plant see a small possibility of having another nuclear power plant in the vicinity. Therefore, a higher salience of nuclear energy does not necessarily mean local employment benefits for them.

The upper panel of *landscape* shows that those who live close to the nuclear power plant do not prefer wind over nuclear even if they attach high importance to landscape (H6a is supported). The lower panel of *landscape* shows that as respondents attach high importance to landscape protection, those living close to wind facilities (between 20 and 80 miles) are more supportive of nuclear over wind. This is in line with our expectation for landscape destruction, which supports Hypothesis 6b.

Lastly, the upper and lower panels of *Supply* show that as respondents attach more importance to stable electricity supply, their support for nuclear over wind increases. We can observe this effect in most areas, except when the distance to the nuclear facility is less than 60 miles and greater than 190 miles, and the distance to the wind facility is less than 30 miles. This could be viewed as partially supporting Hypothesis 7, although its theoretical rationale is not clear. Our findings are summarized in Table 4.

**Table 4:** Summary of hypotheses testing.

Hypothesis	Finding
H1: Washington state will support wind over nuclear.	Supported
H2a: Respondents residing closer to the Richland nuclear facility are less supportive of nuclear over wind.	Not supported
H2b: Respondents residing closer to wind facilities are more supportive of nuclear over wind.	Not supported
H3a: Respondents attaching high importance to health effects of energy facilities support wind over nuclear.	Supported
H3b: Respondents attaching high importance to local jobs support nuclear over wind.	Supported
H3c: Respondents attaching high importance to preserving local landscape support nuclear over wind.	Supported
H3d: Respondents attaching high importance to stable electricity supply support nuclear over wind.	Supported
H4a: Respondents attaching high importance to health issues will prefer wind over nuclear when they live closer to the Richland power plant.	Not supported
H4b: Respondents attaching high importance to health issues will now show a preference for wind over nuclear when they live closer to wind facilities.	Supported
H5a: Respondents attaching high importance to jobs will prefer nuclear over wind when they live closer to the Richland nuclear power plant.	Not supported
H5b: Respondents attaching high importance to jobs will prefer wind over nuclear when they live closer to a wind facility.	Supported
H6a: Respondents attaching high importance to landscape aesthetics will not show a preference for wind over nuclear when they live closer to the Richland nuclear plant.	Supported
H6b: Respondents attaching high importance to landscape aesthetic will prefer nuclear over wind when they live closer to wind facilities.	Supported
H7: Respondents attaching high importance to stable electricity supply will prefer nuclear over wind irrespective of where they live.	Partially supported

## Conclusion

Against a backdrop of an aggressive energy transition plan in Washington State, this study seeks to understand public support for nuclear in relation to wind energy. Drawing on the literature on public support for energy projects, we expected that proximity to existing energy facilities, coupled with the varying importance respondents attach to health, landscape, local jobs, and electricity stability dimensions, influence public support for nuclear over wind. While most studies assessed public support in the context of a single energy source, this study explored how respondents choose between two zero-emission energy sources, which is a more realistic debate in the context of climate change. Furthermore, we systematically explored the role of physical proximity to existing nuclear or wind facilities to understand the drivers of respondents’ support for the state-level energy mix, and how it conditions the importance they attach to health, landscape, local jobs, and electricity stability implications of nuclear and wind energy. Overall, we highlight that the public does not assess the impacts of energy choices on society uniformly.

Our main contributions are two folds. First, we build on the insights of NIMBY/YIMBY in a novel way. As opposed to assessing support for a local project, we explore how support for a state-level energy policy mix might be influenced by respondents’ prior exposure to energy facilities. The intuition is that the benefits and costs of energy projects first-hand might shape individuals’ preferences for energy policy choices. To our surprise, we find that the physical proximity to existing energy facilities does not *directly* shape public support for nuclear in relation to wind. Rather, it *moderates* the effect of health, jobs, landscapes, and supply stability dimensions of nuclear and wind. This suggests that NIMBY/YIMBY insights, originally developed to understand local opposition to unwanted local facilities, are valuable for understanding public support for energy policy choices at the state level.

To put these results in the context of Washington State, existing wind turbines and the nuclear power plant are predominantly located in the rural Eastern part. Thus, the absence of a direct association between proximity to existing energy facilities and preference for energy policy mix suggests that the political urban-rural divide is not reflected in support for state-level energy policy [70–73]. This is an important finding because scholars have noted the emerging rural backlash on climate issues as reflected in the proliferation of restrictive ordinances in rural counties that restrict utility-scale wind and solar projects. Future work should explore how our findings might hold in the context of a proposed energy policy facility in the neighborhood (to test NIMBY/YIMBY effects), as opposed to a hypothetical energy policy mix at the state level.

Second, this paper underlines the importance of examining how individuals evaluate alternative energy sources. Think of the Russian invasion of Ukraine. The debate is on how to reduce the energy (gas) dependence of Europe on Russia. There are several alternatives such as a more aggressive exploration of gas supplies within Europe and imports from the U.S., a higher role for nuclear, as well as a quicker switch over to renewables. Further, public support for any source (say nuclear) is probably influenced not only by the pros and cons of nuclear itself (health impact, local jobs, stable electricity supply) but also by those of other sources which are in play in the policy discussion. For example, concerns about landscape aesthetics associated with wind energy might sway the support of some respondents in favor of nuclear energy. Taken together, public support for energy policy choices is influenced by which different energy sources are under consideration, their pros and cons are framed, and what sort of prior experience individuals might have with any type of energy facility. This suggests that to assess public support for a specific energy mix, policymakers should carefully tailor their communication of the benefits and costs of different energy policy options to different audiences.

Our results are generalizable across the US states (and perhaps across the world). As we noted, renewable energy projects are facing local opposition, even in liberal states. Further, there are 55 commercial nuclear power plants in 28 U.S. states which provides about 20% of U.S. electricity and constitutes about half of emission-free electricity. With increased carbon emissions and the Russian invasion of Ukraine, there is a renewed policy interest in nuclear energy. Recently, the state of California has reversed itself and seeks to extend the regulatory life of the Diablo Canyon plant which is scheduled to be shut down in 2025. Diablo Canyon supplies 15% of California’s carbon-free electricity. Broadly, the regulatory licenses (typically 40 years) of most of America’s nuclear reactors will be expiring soon because they were constructed in the 1970s and 1980s. Yet, it is not clear what is the level of local support (as opposed to national-level opinion polls) to relicense them or construct new plants. Thus, assessing local support for existing nuclear plants is crucial in planning for the clean energy transition at the state and national levels.

This paper has several limitations. First, we have focused on physical proximity to existing nuclear and wind facilities, as opposed *to perceptions* about physical proximity. One might live near a nuclear plant but not notice it. Or one might notice a wind turbine while driving to work and believe that it is located in the neighborhood, although it is situated quite far from the place of residence. Thus, future research should also ask about perceptions of proximity as opposed to focusing on physical proximity alone. Moreover, this research design should be replicated in other states, especially where nuclear energy plays a less visible role in meeting state-level electricity needs.

Second, while we have focused on the nuclear versus wind debate, arguably, other energy sources might be more relevant in different states or countries. Specifically, citizens might debate solar versus wind or nuclear versus gas. Different energy sources have specific pros and cons regarding local jobs, health impact, landscape aesthetics, and supply reliability. It would be instructive to see how our results travel to other energy mixes. This is important for policymaking because, with appropriate media framing, public support for energy sources could be influenced by highlighting the pros and cons, at least at the margin, of energy sources.

## Supporting information

S1 Appendix Appendix for “Comparing public support for nuclear and wind energy in Washington State”. (DOCX)
