# Supplementary material for: Comparing public support for nuclear and wind energy in Washington State
Source: PLoS One. 2023 Apr 26;18(4):e0284208. doi: 10.1371/journal.pone.0284208 (PMC10132544; doi:10.1371/journal.pone.0284208)
Supplement: S1 Appendix — (DOCX) [file pone.0284208.s001.docx]

***Appendix***

***for***

***“Comparing public support for nuclear and wind energy in Washington State”***

**Table of Contents**

**Table A1.** Results of Models 1 and 2 with inattentive respondents

**Table A2.** Full result of Model 1

**Table A3.** Regression table for Model 2

**Text A4.** Full text of survey questions and display instructions

**Table A1. Results of Model 1 and 2 including inattentive respondents**

|  | Model 1 | | | Model 2 | | |
| --- | --- | --- | --- | --- | --- | --- |
|  | Coef. | SE |  | Coef. | SE |  |
| Dist_nuclear | -0.029 | 0.043 |  | 0.174 | 0.282 |  |
| Dist_wind | 0.052 | 0.065 |  | -0.332 | 0.422 |  |
| Health | -6.133 | 1.622 | ^***^ | -7.244 | 6.026 |  |
| Job | -5.161 | 1.892 | ^**^ | -1.606 | 7.656 |  |
| Landscape | 3.510 | 1.495 | ^*^ | 1.799 | 1.799 |  |
| Supply | 8.448 | 2.484 | ^***^ | 9.839 | 9.839 |  |
| Dist_nuclear * Health |  |  |  | -0.005 | 0.046 |  |
| Dist_nuclear * Job |  |  |  | -0.038 | 0.057 |  |
| Dist_nuclear * Landscape |  |  |  | 0.022 | 0.043 |  |
| Dist_nuclear * Supply |  |  |  | -0.033 | 0.072 |  |
| Dist_wind * Health |  |  |  | 0.034 | 0.067 |  |
| Dist_wind * Job |  |  |  | 0.038 | 0.075 |  |
| Dist_wind * Landscape |  |  |  | -0.028 | 0.063 |  |
| Dist_wind * Supply |  |  |  | 0.063 | 0.098 |  |
| Trust_reg | 5.020 | 2.270 | ^*^ | 5.080 | 2.287 | ^*^ |
| Trust_health | -10.669 | 1.957 | ^***^ | -10.711 | 1.970 | ^***^ |
| Trust_plant | 17.605 | 2.061 | ^***^ | 17.525 | 2.078 | ^***^ |
| Env | -18.110 | 3.879 | ^***^ | -17.896 | 3.914 | ^***^ |
| Urban_rural | 5.096 | 4.778 |  | 5.523 | 4.844 |  |
| Male | 8.697 | 3.223 | ^**^ | 8.696 | 3.238 | ^**^ |
| Age (Year of Birth) |  |  |  |  |  |  |
| 1946 to 1964 | -13.815 | 8.146 | ^+^ | -13.749 | 8.190 | ^+^ |
| 1965 to 1996 | -19.976 | 8.077 | ^*^ | -20.045 | 8.123 | ^*^ |
| After 1996 | -14.038 | 9.103 |  | -14.051 | 9.172 |  |
| Income |  |  |  |  |  |  |
| $40,000 - $69,999 | -7.797 | 3.979 |  | -0.599 | 4.024 |  |
| $70,000 - $104,999 | -4.013 | 4.341 |  | -3.938 | 4.383 |  |
| $105,000 - $159,999 | -8.653 | 5.210 | ^+^ | -8.361 | 5.255 |  |
| $160,000 - | 5.026 | 6.514 |  | 5.235 | 6.568 |  |
| Education |  |  |  |  |  |  |
| High school (incomplete) | 90.257 | 44.608 | ^*^ | 89.769 | 44.805 | ^*^ |
| High school | 97.594 | 43.666 | ^*^ | 96.892 | 43.845 | ^*^ |
| College (no degree) | 94.345 | 43.602 | ^*^ | 93.605 | 43.738 | ^*^ |
| 4 yrs. college | 94.039 | 43.622 | ^*^ | 93.404 | 43.805 | ^*^ |
| Grad. school | 99.946 | 43.642 | ^*^ | 99.217 | 43.823 | ^*^ |
| Race |  |  |  |  |  |  |
| Asian | 1.365 | 9.024 |  | 1.775 | 9.102 |  |
| Caucasian | -3.321 | 7.510 |  | -3.083 | 7.560 |  |
| Latin or Hispanic | 7.657 | 9.336 |  | 7.752 | 9.451 |  |
| Native American | -6.927 | 12.579 |  | -6.505 | 12.702 |  |
| Native Hawaiian / Pacific Islander | 39.331 | 20.801 | ^+^ | 39.215 | 20.946 | ^+^ |
| Other/Unknown | 0.365 | 13.008 |  | 0.858 | 13.080 |  |
| Religion |  |  |  |  |  |  |
| Buddhist | -6.063 | 12.926 |  | -5.796 | 12.991 |  |
| Catholic | 0.936 | 4.636 |  | 0.920 | 4.669 |  |
| Hindu | -22.875 | 21.982 |  | -23.083 | 22.081 |  |
| Jewish | 13.972 | 13.481 |  | 14.002 | 13.583 |  |
| Muslim | 1.535 | 13.035 |  | 1.844 | 13.252 |  |
| Orthodox | -20.039 | 18.104 |  | -19.369 | 18.307 |  |
| Protestant | 6.498 | 4.492 |  | 6.381 | 4.521 |  |
| Other Christians | 2.173 | 4.245 |  | 2.402 | 4.280 |  |
| Other faiths | -0.525 | 7.024 |  | -0.403 | 7.094 |  |
| Party Identification |  |  |  |  |  |  |
| Democrat | -6.546 | 4.242 |  | -6.922 | 4.305 |  |
| Independent | 1.208 | 4.107 |  | 0.920 | 4.148 |  |
| Intercept | -85.993 | 46.277 | ^+^ | -94.833 | 56.955 | ^+^ |
| *N* | 870 | | | 870 | | |
| Adj. *R*^2^ | 0.318 | | | 0.312 | | |
| Note: ^+^: p < 0.1; ^*^: p < 0.05; ^**^: p < 0.01; ^***^: p<0.001 | | | | | | |

**Table A2. Full result of Model 1**

|  | Model 1 | | | Model 1 + Region | | |
| --- | --- | --- | --- | --- | --- | --- |
|  | Coef. | SE |  | Coef. | SE |  |
| Dist_nuclear | -0.029 | 0.044 |  | -0.009 | 0.051 |  |
| Dist_wind | 0.063 | 0.066 |  | 0.072 | 0.066 |  |
| Health | -5.896 | 1.649 | ^***^ | -5.791 | 1.655 | ^***^ |
| Job | -5.159 | 1.916 | ^**^ | -5.057 | 1.920 | ^**^ |
| Landscape | 4.205 | 1.515 | ^**^ | 4.154 | 1.516 | ^**^ |
| Supply | 9.262 | 2.514 | ^***^ | 9.259 | 2.514 | ^***^ |
| Trust_reg | 4.370 | 2.309 | ^+^ | 4.443 | 2.311 | ^+^ |
| Trust_health | -10.090 | 2.016 | ^***^ | -10.086 | 2.016 | ^***^ |
| Trust_plant | 17.889 | 2.096 | ^***^ | 17.854 | 2.097 | ^***^ |
| Env | -17.761 | 3.940 | ^***^ | -17.602 | 3.946 | ^***^ |
| Urban_rural | 4.818 | 4.783 |  | 4.312 | 4.827 |  |
| Male | 8.901 | 3.262 | ^**^ | 9.113 | 3.273 | ^**^ |
| Age (Year of Birth) |  |  |  |  |  |  |
| 1946 to 1964 | -13.570 | 8.253 |  | -13.928 | 8.268 | ^+^ |
| 1965 to 1996 | -18.840 | 8.210 | ^*^ | -19.042 | 8.216 | ^*^ |
| After 1996 | -15.426 | 9.318 | ^+^ | -15.522 | 9.321 | ^+^ |
| Income |  |  |  |  |  |  |
| $40,000 - $69,999 | -0.485 | 4.029 |  | -0.293 | 4.038 |  |
| $70,000 - $104,999 | -3.851 | 4.400 |  | -3.545 | 4.419 |  |
| $105,000 - $159,999 | -9.189 | 5.253 | ^+^ | -8.774 | 5.281 | ^+^ |
| $160,000 - | 1.055 | 6.577 |  | 1.674 | 6.626 |  |
| Education |  |  |  |  |  |  |
| High school (incomplete) | 91.985 | 44.529 | ^*^ | 90.244 | 44.594 | ^*^ |
| High school | 98.998 | 43.602 | ^*^ | 97.953 | 43.633 | ^*^ |
| College (no degree) | 95.667 | 43.535 | ^*^ | 94.399 | 43.575 | ^*^ |
| 4 yrs. college | 96.635 | 43.558 | ^*^ | 95.315 | 43.600 | ^*^ |
| Grad. school | 102.391 | 43.585 | ^*^ | 101.129 | 43.625 | ^*^ |
| Race |  |  |  |  |  |  |
| Asian | -2.219 | 9.362 |  | -2.229 | 9.364 |  |
| Caucasian | -4.055 | 7.887 |  | -4.350 | 7.898 |  |
| Latin or Hispanic | 6.065 | 9.743 |  | 6.065 | 9.745 |  |
| Native American | -12.065 | 13.007 |  | -12.766 | 13.040 |  |
| Native Hawaiian / Pacific Islander | 38.709 | 20.886 | ^+^ | 38.229 | 20.900 | ^+^ |
| Other/Unknown | -1.365 | 13.189 |  | -1.496 | 13.193 |  |
| Religion |  |  |  |  |  |  |
| Buddhist | -5.204 | 12.905 |  | -5.234 | 12.908 |  |
| Catholic | 1.707 | 4.738 |  | 1.451 | 4.750 |  |
| Hindu | -21.802 | 21.943 |  | -21.658 | 21.949 |  |
| Jewish | 13.529 | 13.465 |  | 13.061 | 13.482 |  |
| Muslim | 10.819 | 13.601 |  | 11.000 | 13.606 |  |
| Orthodox | -14.116 | 21.801 |  | -14.080 | 21.806 |  |
| Protestant | 6.347 | 4.510 |  | 6.287 | 4.512 |  |
| Other Christians | 2.163 | 4.294 |  | 2.104 | 4.296 |  |
| Other faiths | 0.743 | 7.102 |  | 0.724 | 7.104 |  |
| Party Identification |  |  |  |  |  |  |
| Democrat | -7.371 | 4.302 | ^+^ | -7.316 | 4.304 | ^+^ |
| Independent | 1.092 | 4.164 |  | 1.217 | 4.168 |  |
| Western Washington |  |  |  | -3.574 | 4.531 |  |
| Intercept | -93.862 | 46.343 | ^*^ | -94.181 | 46.355 | ^*^ |
| *N* | 844 | | | 844 | | |
| Adj. *R*^2^ | 0.323 | | | 0.323 | | |
| Note: ^+^: p < 0.1; ^*^: p < 0.05; ^**^: p < 0.01; ^***^: p<0.001 | | | | | | |

**Table A3. Regression table for Model 2**

|  | Coef. | SE |  |
| --- | --- | --- | --- |
| Dist_nuclear | 0.286 | 0.295 |  |
| Dist_wind | -0.145 | 0.428 |  |
| Health | -4.964 | 6.126 |  |
| Job | -0.571 | 7.937 |  |
| Landscape | 2.213 | 5.539 |  |
| Supply | 11.159 | 9.882 |  |
| Dist_nuclear * Health | -0.022 | 0.047 |  |
| Dist_nuclear * Job | -0.045 | 0.058 |  |
| Dist_nuclear * Landscape | 0.020 | 0.044 |  |
| Dist_nuclear * Supply | -0.041 | 0.074 |  |
| Dist_wind * Health | 0.043 | 0.068 |  |
| Dist_wind * Job | 0.041 | 0.075 |  |
| Dist_wind * Landscape | -0.020 | 0.064 |  |
| Dist_wind * Supply | 0.073 | 0.099 |  |
| Trust_reg | 4.487 | 2.328 | ^+^ |
| Trust_health | -10.150 | 2.030 | ^***^ |
| Trust_plant | 17.791 | 2.116 | ^***^ |
| Env | -17.471 | 3.975 | ^***^ |
| Urban_rural | 5.152 | 4.846 |  |
| Male | 8.828 | 3.278 | ^**^ |
| Age (Year of Birth) |  |  |  |
| 1946 to 1964 | -13.665 | 8.301 |  |
| 1965 to 1996 | -19.071 | 8.256 | ^*^ |
| After 1996 | -15.893 | 9.397 | ^+^ |
| Income |  |  |  |
| $40,000 - $69,999 | -0.320 | 4.074 |  |
| $70,000 - $104,999 | -3.844 | 4.441 |  |
| $105,000 - $159,999 | -8.857 | 5.302 | ^+^ |
| $160,000 - | 1.261 | 6.636 |  |
| Education |  |  |  |
| High school (incomplete) | 92.075 | 44.725 | ^*^ |
| High school | 94.990 | 43.775 | ^*^ |
| College (no degree) | 96.188 | 43.710 | ^*^ |
| 4 yrs. college | 101.760 | 43.736 | ^*^ |
| Grad. school |  |  |  |
| Race |  |  |  |
| Asian | -1.638 | 9.453 |  |
| Caucasian | -3.632 | 7.945 |  |
| Latin or Hispanic | 6.790 | 9.881 |  |
| Native American | -12.040 | 13.144 |  |
| Native Hawaiian / Pacific Islander | 38.812 | 21.032 | ^+^ |
| Other/Unknown | -0.543 | 13.264 |  |
| Religion |  |  |  |
| Buddhist | -4.815 | 12.967 |  |
| Catholic | 1.717 | 4.770 |  |
| Hindu | -21.983 | 22.038 |  |
| Jewish | 13.450 | 13.566 |  |
| Muslim | 12.371 | 13.876 |  |
| Orthodox | -12.785 | 22.079 |  |
| Protestant | 6.171 | 4.540 |  |
| Other Christians | 2.512 | 4.331 |  |
| Other faiths | 0.699 | 7.182 |  |
| Party Identification |  |  |  |
| Democrat | -7.782 | 4.365 | ^+^ |
| Independent | 0.814 | 4.207 |  |
| Intercept | -114.314 | 57.962 | ^*^ |
| *N* | 844 | | |
| Adj. *R*^2^ | 0.318 | | |
| Note: ^+^: p < 0.1; ^*^: p < 0.05; ^**^: p < 0.01; ^***^: p<0.001 | | | |

**Text A4. Full text of survey questions and display instructions**

**Welcome page**

Welcome to this survey! We are very grateful for your participation.

This survey focuses on energy policy in the state of Washington and should take around 8 minutes to complete. It is solely for scientific purposes and has no commercial or government-related purpose. 

The survey is anonymous. The information you provide will not be stored or used in any way that could reveal your personal identity. There are no known risks posed by participating in this survey. The survey has been reviewed by University of Washington's Human Subject Division (STUDY00014049). Participation is voluntary, and you may withdraw your participation at any time.

Our research will only produce meaningful results if you read and think about each question carefully and express your true personal opinion. Please read the questions carefully and answer to the best of your knowledge.

[Page break]

Please read the following text on two pages **very carefully** and then answer the question.

*TransAlta Centralia coal power plant in Washington state will be closing down the second unit in 2025. Some are also advocating for a removal of four hydroelectric dams on Snake River that impede salmon migration.*

*The state of Washington is also gaining population, which will increase demand for energy. Finally, Washington state plans to ban the sale of new gasoline-powered cars by the end of this decade. Thus, electricity demand will also increase with the electrification of the transportation sector.*

*The question is, how would electricity demand in the future be met?* ***Because Washington state wants to achieve the status of net zero carbon emissions by 2050, new electricity facilities will rely on non-carbon emitting facilities.*** *There are two major technologies that Washington state can expand:* ***wind and nuclear****.*

*Washington state already has a nuclear plant in Richland, Eastern Washington. New nuclear capacity could be located within this plant. Washington State also relies on wind energy, generated by wind farms, such as Windy Point/Windy Flats Project. Additional wind farms can be built in Eastern Washington.*

[Page break]

**Attention check**

We want to make sure that you are paying attention to the information provided in this survey. Based on the information provided above, please respond to the following.

1. Washington State wants to discontinue

- Coal energy
- Solar energy
- Wind energy

2. Hydroelectric dams that are proposed to be removed are on the following river

- Columbia River
- Snake River
- Snoqualmie River

1. Washington aims to have net zero emission electricity by

- 2040
- 2050
- 2060

[Page break]

*Both sources have pros and cons:*

|  | **Pros** | **Cons** |
| --- | --- | --- |
| Nuclear | - Generates electricity whenever needed (24 hours and 7 days a week) - A large number of permanent local jobs | - Communities living near nuclear plants face a radiation risk - Nuclear waste needs to be stored safely |
| Wind | - Farmers and rural landowners can earn income by leasing their land to wind farms - New jobs, especially during the construction. | - Wind turbines clash with the rural landscape, which could hurt land values - Electricity generated only when there is wind |

New, non-carbon emitting electricity generation capacity of about 4,000 MW will have to be installed in Washington State. This will be met by **wind and nuclear**. How much of this should be nuclear and how much wind? Please move the sliders so that the combination of nuclear and wind is **equal to 100**. Note that you cannot select a value that makes the combination over 100.

Wind 0 10 20 30 40 50 60 70 8 0 90 100%

Nuclear 0 10 20 30 40 50 60 70 8 0 90 100%

*Respondents receive an error message and cannot move on to the next question if the combination of nuclear and wind is not equal to 100.

[Page break]

**Mechanism questions**

Q. Criteria

Please reflect on your response. How ***important*** were the following factors for your decision on the electricity generation?

- I want reliable electricity supply 24/7.
- I want new jobs or economic benefits to be created in my community.
- I do not want electricity generation and its waste to harm human health.
- I do not want new electricity facilities clash with the rural landscape.

1. Not at all important
2. Slightly important
3. Fairly important
4. Very important

[Page break]

Q. Nuclear plant safety

Generally speaking, do you think nuclear power plants are safe or not safe?

1. Safe
2. Not safe

Q. Media portrayal of nuclear energy

In your opinion, how does the U.S. media portray nuclear energy?

1. In a balanced way, it presents risks and benefits of nuclear technology.
2. It focuses on the risks of nuclear energy to human health and the environment.
3. It focuses on promises of new, modular nuclear technologies.
4. Don’t know.

[Page break]

Q. Trust in nuclear energy regulators

How much do you trust the following:

| The federal Nuclear Regulatory Commission | 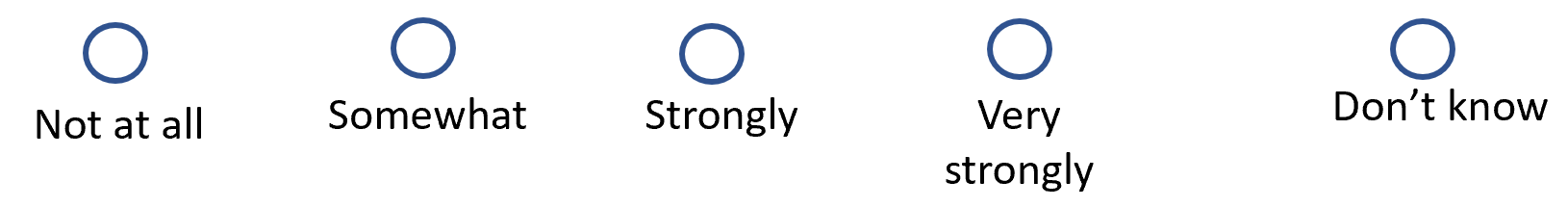 |
| --- | --- |
| Washington State Department of Health | 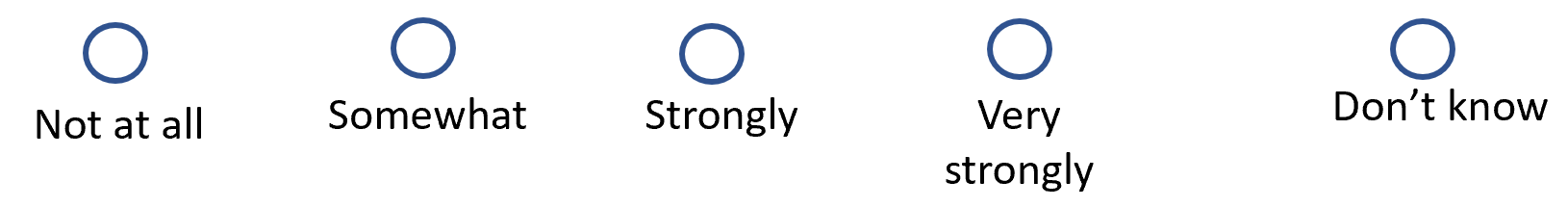 |
| Nuclear plant operators | 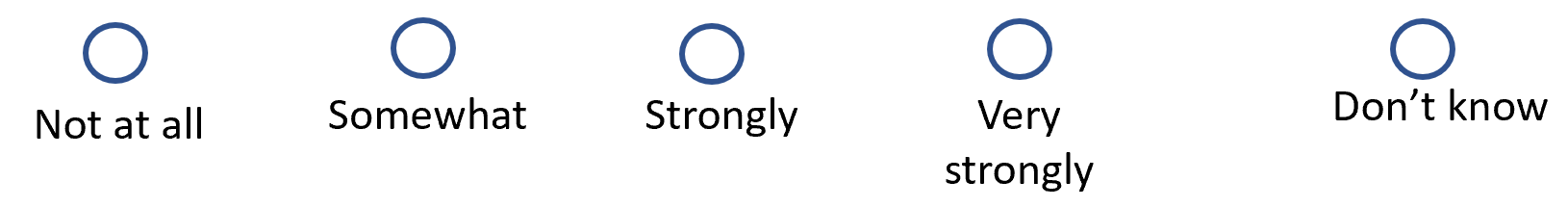 |

Q. Risk perception

If a nuclear accident occurred, it would have a negative impact (life, health, or economic loss) on myself and my family. (*Directions of choices are switched)

1. Agree
2. Somewhat agree
3. Neither agree nor disagree
4. Somewhat disagree
5. Disagree
6. Don’t know

[Page break]

Q. Environmental Attitude (from WVS)

Here are two statements people sometimes make when discussing the environment and economic growth. Which of them comes closer to your own point of view?

A. Protecting the environment should be given priority, even if it causes slower economic growth and some loss of jobs.

B. Economic growth and creating jobs should be the top priority, even if the environment suffers to some extent.

(1) Statement A

(2) Statement B

Q. Electricity cost

Nuclear energy would increase my electricity bill more than wind energy.

1. Agree
2. Neither agree nor disagree
3. Disagree
4. Don’t know

[Page break]

Q. How much do you agree or disagree with the following statements?

1. Wind turbines are troublesome for local residents because they make noise and destroy landscape.

2. Wind turbines are troublesome for local people because they cause electromagnetic interference and their blades can fall on people when they are damaged by a storm.

3. Wind turbines are troublesome for local people because they are harmful to birds and other wild life.

1. Agree
2. Somewhat agree
3. Neither agree nor disagree
4. Somewhat disagree
5. Disagree
6. Don’t know

Q. Genetically modified foods

Do you support a ban on genetically modified foods? [Randomly change the order of No and Yes]

(1) No

(2) Yes

(3) Don’t know

[Page break]

**Demographic questions**

Q. Age

When were you born? [Provide pull-down menu]

1. Before 1946
2. 1946-1964
3. 1965-1996
4. After 1996
5. Prefer not to answer

Q. Gender

What is your gender? [Provide pull-down menu]

(1) Male

(2) Female

(3) Non-binary

(4) Prefer not to answer

[Page break]

Q. Race/ethnicity

Please specify your race/ethnicity. You can select more than one.

1. African-American
2. Caucasian
3. Latino or Hispanic
4. Asian
5. Native American
6. Native Hawaiian or Pacific Islander
7. Other/Unknown
8. Prefer not to answer.

Q. Religion

What is your religious preference?

1. Protestant
2. Catholic
3. Orthodox
4. Other Christians
5. Jewish
6. Muslim
7. Hindu
8. Buddhist
9. Other faiths
10. Not religious
11. Don’t know/prefer not to say

[Page break]

Q. Location

Which ZIP code do you currently live in?

XXXXX

*If XXXXX is not a Washington ZIP code → End of the survey

Q. Education

What best describes the highest level of education you completed?

1. Middle School or below
2. High school incomplete
3. High school
4. Some college, no degree
5. Four-year college
6. Graduate school

[Page break]

Q. Employment status

Are you currently employed?

(1) Yes (this includes self-employment)

(2) No, but I am actively looking for a job

(3) No, I am a student

(4) No, I am retired

(5) No, I am taking care of the household

(6) Other

Q. Household income

What is your annual ***household*** income before taxes? Like the rest of the survey, this question is completely confidential and will be used only to classify the survey responses. [Provide pull-down menu]

(1) Less than $40,000

(2) $40,000 - $69,999

(3) $70,000 - $104,999

(4) $105,000 - $159,999

(5) $160,000 or higher

(6) Prefer not to answer

[Page break]

Q. Political affiliation

In the U.S., many people lean towards a particular party, although they may occasionally vote for a candidate from a different party. How about you; do you in general lean towards a particular party? If so, which one?

[Randomize order of items]

(1) Republican

(2) Democrat

(3) Independent

(4) Prefer not to answer

[Page break]

We appreciate your participation.
